# Genetic data and climate niche suitability models highlight the vulnerability of a functionally important plant species from south‐eastern Australia

**DOI:** 10.1111/eva.12958

**Published:** 2020-04-17

**Authors:** Adam D. Miller, Craig Nitschke, Andrew R. Weeks, William L. Weatherly, Simon D. Heyes, Steve J. Sinclair, Owen J. Holland, Aggie Stevenson, Linda Broadhurst, Susan E. Hoebee, Craig D. H. Sherman, John W. Morgan

**Affiliations:** ^1^ Centre for Integrative Ecology School of Life and Environmental Sciences Deakin University Geelong Vic Australia; ^2^ Deakin Genomics Centre Deakin University Geelong Vic Australia; ^3^ School of Ecosystem and Forest Sciences The University of Melbourne Richmond Vic Australia; ^4^ School of BioSciences The University of Melbourne Parkville Vic Australia; ^5^ Friends of the Forgotten Woodlands Dunkeld Vic Australia; ^6^ Department of Ecology, Environment and Evolution La Trobe University Bundoora Vic Australia; ^7^ Department of Environment, Land, Water and Planning Arthur Rylah Institute Heidelberg Vic Australia; ^8^ Glenelg Hopkins Catchment Management Authority Hamilton Vic Australia; ^9^ Centre for Australian National Biodiversity Research CSIRO National Research Collections Canberra ACT Australia

**Keywords:** *Banksia marginata*, climate change, climate niche, evolutionary potential, genetic rescue, habitat fragmentation, population genetics

## Abstract

Habitat fragmentation imperils the persistence of many functionally important species, with climate change a new threat to local persistence due to climate niche mismatching. Predicting the evolutionary trajectory of species essential to ecosystem function under future climates is challenging but necessary for prioritizing conservation investments. We use a combination of population genetics and niche suitability models to assess the trajectory of a functionally important, but highly fragmented, plant species from south‐eastern Australia (*Banksia marginata*, Proteaceae). We demonstrate significant genetic structuring among, and high level of relatedness within, fragmented remnant populations, highlighting imminent risks of inbreeding. Population simulations, controlling for effective population size (*N*_e_), suggest that many remnant populations will suffer rapid declines in genetic diversity due to drift in the absence of intervention. Simulations were used to demonstrate how inbreeding and drift processes might be suppressed by assisted migration and population mixing approaches that enhance the size and connectivity of remnant populations. These analyses were complemented by niche suitability models that predicted substantial reductions of suitable habitat by 2080; ~30% of the current distribution of the species climate niche overlaps with the projected distribution of the species climate niche in the geographic region by the 2080s. Our study highlights the importance of conserving remnant populations and establishing new populations in areas likely to support *B. marginata* in the future, and adopting seed sourcing strategies that can help populations overcome the risks of inbreeding and maladaptation. We also argue that ecological replacement of *B. marginata* using climatically suited plant species might be needed in the future to maintain ecosystem processes where *B. marginata* cannot persist. We recommend the need for progressive revegetation policies and practices to prevent further deterioration of species such as *B. marginata* and the ecosystems they support.

## 1 INTRODUCTION

Global climate change poses a direct threat to plant communities and the ecosystems they support (Bongaarts, 2019). Climate change is predicted to disrupt the adaptedness of local plant populations, ultimately leading to changes in species distributions and community composition (Rehfeldt & Jaquish, 2010). Some plant communities in Australia are already showing signs of climate stress; low rainfall and elevated temperatures are responsible for significant dieback in mangrove forests (Duke et al., 2017) and eucalypt species (Brouwers, Matusick, Ruthrof, Lyons, & Hardy, 2013), while fire (Fairman, Bennett, Tupper, & Nitschke, 2017; Steel, Fontaine, Ruthrof, Burgess, & Hardy, 2019), rising temperatures and reduced snow cover are causing shifts in community assemblages in alpine regions (Camac, Williams, Wahren, Hoffmann, & Vesk, 2017; Wahren et al., 2013). A significant challenge for conservation planning is to identify those species most at risk of such changes and to implement actions that maximize their adaptability (Levin et al., 2013).

Climate change is occurring at rates faster than plant species have been able to accommodate historically via migration (Loarie et al., 2009). For many plant species, migration is compromised by dispersal limitation and habitat discontinuities. Consequently, these species will become increasingly dependent on combatting climate change via in situ adaptation (Hoffmann & Sgro, 2011). This will be particularly challenging for sexually reproducing species persisting in highly modified landscapes, where the genetic integrity and adaptability of isolated remnant populations can be compromised by genetic drift and inbreeding effects, and dependency on local genotypes that may be maladapted to future climates (Frankham, 2015; Hoffmann, Sgro, & Kristensen, 2017). In such cases, intervention may be necessary to broaden the genetic basis of remnant plant populations, to enhance their adaptability and to mitigate the deleterious effects of inbreeding (Miller et al., 2019; Weeks et al., 2011).

The intentional movement of genotypes among populations and across environmental gradients is now being advocated to address such risks (Kelly & Phillips, 2016; Sgro, Lowe, & Hoffmann, 2011; Whiteley, Fitzpatrick, Funk, & Tallmon, 2015), with successful outcomes being increasingly reported (Bossuyt, 2007; Frankham, 2015; Poirier, Coltman, Pelletier, Jorgenson, & Festa‐Bianchet, 2019; Weeks et al., 2017). Where inbreeding risks are significant (i.e. small, isolated populations), or already quantifiable (i.e. there is evidence of reduced fitness and genetic load), assisted migration of small numbers of individuals per generation into populations can effectively reduce the detrimental consequences of inbreeding (Frankham, 2015; Weeks et al., 2011). Where future climate adaptability of remnant populations is the priority, moving individuals from current warm, dry‐adapted populations to colder, wetter locations may increase the probability of future climate adaptation (Hoffmann & Sgro, 2011). This approach may primarily apply to species with wide altitudinal or latitudinal ranges, many of which show genetically based clines across thermal and aridity gradients (Aitken & Bemmels, 2016; Halbritter et al., 2018; Jeffery et al., 2017; Pereira, Sasaki, & Burton, 2017). For the restoration of threatened plant species, these advances have led to restoration strategies that move beyond the exclusive use of local provenance, advocating broad genetic sampling and inclusion of nonlocal and climate‐matched genotypes (Breed, Stead, Ottewell, Gardner, & Lowe, 2013; Broadhurst et al., 2008; Prober et al., 2015).

While opportunities may exist to bolster the genetic basis of plant populations, consideration needs to be given to species' climate niches under future climates. Species distribution models suggest that major shifts in suitable habitat will occur under climate change. In some cases, it has been suggested that there will be no (or minimal) overlap between current and future predicted climatic niches (Ledig, Rehfeldt, & Jaquish, 2012; Rehfeldt & Jaquish, 2010; Wang, Wang, Innes, Nitschke, & Kang, 2016). This information is critical for directing management towards the conservation of remnant populations persisting in areas more likely to support the species under climate change, and considering interventions, such as assisted range expansion, to facilitate species movement to climatically suitable areas (Cole et al., 2011; Hoegh‐Guldberg et al., 2008; Wadgymar, Cumming, & Weis, 2015; Winder, Nelson, & Beardmore, 2011). Additionally, ecological replacement may be necessary, involving the substitution of a species with a climatically matched and functionally equivalent species in areas where the fundamental niche of a species is likely to be exceeded under climate change (Doherty, Lavorel, Colloff, Williams, & Williams, 2017; Lunt et al., 2013; Nitschke & Innes, 2008).

Predicting the evolutionary trajectory of species essential to ecosystem function under future climates is challenging but necessary for prioritizing conservation investments. In this context, we use a combination of population genetic and climate modelling tools to assess the likely evolutionary trajectory of a functionally important plant species from south‐eastern Australia. The Silver Banksia (*Banksia marginata*, Proteaceae) is a species of savannas and forests that was once widespread, but has suffered significant decline over the last century. The species is now largely limited to small remnants in highly modified landscapes and is showing signs of climate stress. Population genetic analyses on remnant tree‐form populations spanning the Victorian Volcanic Plains region of western Victoria provide insights into patterns of contemporary gene flow and genetic structure. We use population simulations to assess risks of declining genetic diversity in small remnants, and discuss management interventions orientated towards enhancing connectivity and genetic diversity to reduce risks of inbreeding and maladaptation under climate change. We complement these analyses with climate niche modelling to investigate likely shifts in suitable habitat within and beyond the current distribution of *Banksia marginata*, providing a framework for conserving existing populations, establishing new populations, considering ecological replacement and identifying climate‐matched seed sources for restoration. We argue that studies of this nature are key to identifying the risk posed by habitat fragmentation and climate change to plant species, and management actions that foster resilience and improved biodiversity outcomes under climate change.

## 2 METHODS

### 2.1 Study species

*Banksia marginata* (Proteaceae) is endemic to forest, savanna and heathlands of south‐eastern Australia. The species varies widely in habit, ranging from a small shrub (<1 m) to a large tree (~12 m), although there is some uncertainty surrounding the taxonomic status of the different growth forms (Collins & George, 2008). The species is primarily outcrossing, but is capable of selfing, and has a long flowering season extending from February to July (Vaughton & Ramsey, 2006). It is a major source of floral nectar for honeyeaters, possums and insects (George, 1984). The woody infructescences are nonserotinous (unlike some other *Banksia* species), with seeds being retained within the follicles for ~12 months prior to dispersal. Seed dispersal occurs predominantly by gravity, with seeds falling near the maternal plant; however, some occasional long‐distance dispersal is thought possible by cockatoos, which have been observed to carry cones (anecdotal observations, Friends of the Forgotten Woodlands). In contrast, inflorescences appear adapted to pollination by mobile vertebrates and invertebrates; this is thought to promote gene flow in other *Banksia* species (Cunningham, 1991; Thavornkanlapachai, Byrne, Yates, & Ladd, 2019).

*Banksia marginata* has a broad distribution extending from northern New South Wales, southwards into Victoria and South Australia, as well as across Tasmania, with remnant populations persisting on the major islands of Bass Strait (Collins & George, 2008). Early reports and surveyor's maps show that *B. marginata* was once scattered but widespread, as a dominant or co‐dominant species in savanna‐like ecosystems (Hateley, 2010; Howitt, 1855; Sinclair & Atchison, 2012). These savannah ecosystems are now either locally extinct or verging on extinction due to extensive land clearance for agriculture (Sinclair & Atchison, 2012). Most remnant *B. marginata* populations are now confined to road and rail verges, and small parcels of land that have escaped intensive land use, such as cemeteries, town commons and remote corners of private agricultural properties; this syndrome of tenuous persistence is common to many plants persisting in Australian agricultural landscapes (Lunt & Spooner, 2005). Remnant populations are typically small (1 to ~800 individuals) and isolated. There is evidence for recruitment bottlenecks in small remnant populations, with some populations failing to set seed (Heyes, Sinclair, Hoebee, & Morgan, 2019). Anecdotal evidence indicates that *B. marginata* is showing signs of climate stress, with both drought and thermal stress driving notable dieback in populations across the species range (Friends of the Forgotten Woodlands, personal communication).

### 2.2 Sampling remnants from a heavily fragmented landscape

**FIGURE 1:**
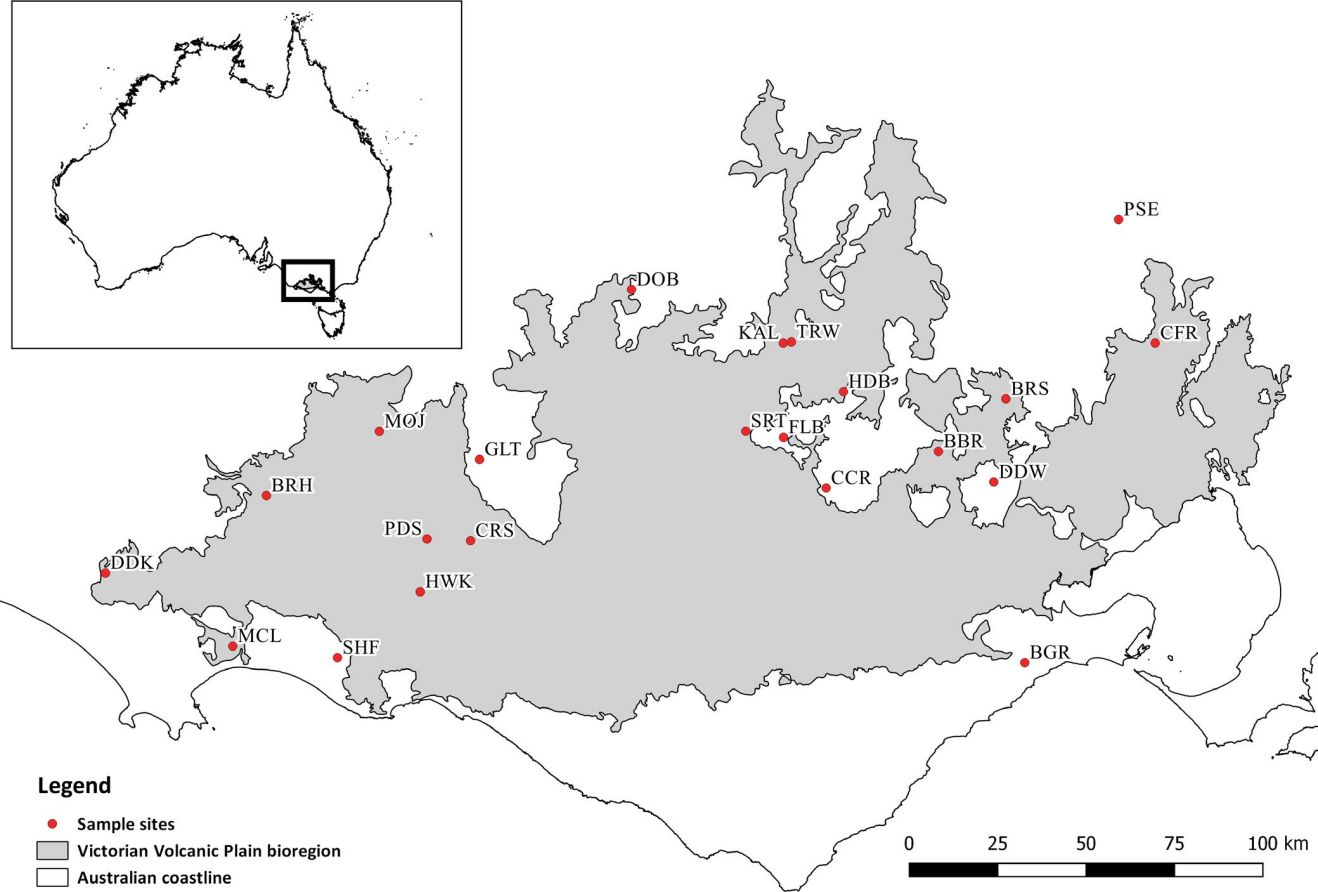
Map of *Banksia marginata* collection sites from the Victorian Volcanic Plains. Refer to Table 1 for site codes

A total of 22 remnant *B. marginata* tree‐form stands from the Victorian Volcanic Plains were selected for genetic analysis (Table 1; Figure 1). Remnants were selected to maximize the geographical spread of sampling across the region and targeted the largest remnants in order to achieve adequate sample sizes for population genetic analysis. Leaf tissue was collected between December 2015 and October 2016, with a maximum of 30 ~ one‐gram samples of fresh growth collected from individual trees from each site. Where possible, sampling adjacent trees was avoided to reduce possible sampling of close relatives. Individual samples with unique identifiers were preserved in paper coffee filters and desiccated with silica gel, and stored in a dried state at room temperature until analysis.

**TABLE 1:** Site location information and corresponding codes, sample sizes (*n*) and approximate census remnant population size for each of the 22 collection sites for *Banksia marginata* from the Victorian Volcanic Plains. Remnant size refers to the number of reproductively mature stems, and does not consider potential for clonality/plant suckers

Site	Site code	Latitude	Longitude	*n*	Remnant size
Bluebridge Road Elaine	BBR	−37.7433	144.0252	25	537
Mt Duneed	BGR	−38.2829	144.2974	25	50
Branxholme Railside	BRH	−37.8183	141.8641	24	~25
Ballan Railside	BRS	−37.6107	144.2431	25	~80
Cape Clear Rokewood Road	CCR	−37.8328	143.6632	25	84
Clarkefield railside.	CFR	−37.4707	144.7220	25	25
Caramut Roadside	CRS	−37.9485	142.5162	25	127
Drik Drik	DDK	−38.0009	141.3377	24	100s
Durdidwarrah	DDW	−37.8220	144.2021	25	>500
Dobie	DOB	−37.3187	143.0507	24	114
Francis Lane	FLB	−37.7022	143.5288	25	143
Glenthompson	GLT	−37.7423	142.5517	24	>100
Haddon	HDB	−37.5881	143.7231	26	69
Hawkesdale/Coltons Road	HWK	−38.0753	142.3495	18	~1,300
Kayleys Lane	KAL	−37.4622	143.5326	16	28
Mount Clay	MCL	−38.1989	141.7394	21	100s
Moutajup	MOJ	−37.6641	142.2329	25	67
Purdeet Siding	PDS	−37.9411	142.3761	5	15
Pastoria East	PSE	−37.1557	144.6067	25	100s
St Helens Flora Reserve	SHF	−38.2367	142.0764	18	20
Skipton Rail Trail	SRT	−37.6851	143.4078	22	22
Trawalla East	TRW	−37.4596	143.5584	25	48

Note

Remnant size refers to the number of reproductively mature stems and does not consider potential for clonality/plant suckers.

### 2.3 Sampling remnants from less modified landscapes

We tested for inherent limitations to gene flow and propagule movement in *B. marginata* by assessing patterns of genetic spatial autocorrelation within large populations (100s of individuals) occurring in extensive and largely intact native habitats. To test for spatial autocorrelation, we genotyped between 80 and 100 individual plants, sampled from 8 to 10 sites (10 trees per site) separated by up to 2.2 km, at three locations from western Victoria: Claude Austin Reserve, Victoria Valley (Grampians National Park) and Durdidwarrah (Barwon Catchment) (Table S1). Field collections occurred in December 2018, with sampling procedures and sample preservation following the protocols previously described.

### 2.4 DNA extraction and genotyping

Genomic DNA was extracted from 30‐mg tissue samples for individual specimens using the NucleoSpin^®^ 96 Plant II protocol (Macherey‐Nagel Inc.), and DNA quantitation was performed as per the QuantiFluor^®^ dsDNA System (Promega Inc). *Banksia marginata* DNA samples were genotyped at 10 microsatellite loci using a composite of genetic markers developed by He, Krauss, & Lamont, 2008 and Fatemi, Houliston, Haddadchi, & Gross, 2013, and additional markers were developed in the present study using the approach outlined in Miller et al., 2019) (Table S2). In order to distinguish PCR products upon capillary separation, primers for the ten microsatellite markers were tagged with a unique fluorescent label during PCR using the method outlined in Blacket, Robin, Good, Lee, and Miller (2012). Reaction matrices for PCR amplification consisted of 5 μl Qiagen multiplex mix (Qiagen), 4 μl of primer mix (0.2 μM of each primer) and 2 μl of template DNA. PCR conditions consisted of an initial 15‐min denaturing step at 94°C, followed by 40 cycles of 94°C for 30 s, 59°C for 1:30 min and 72°C for 1:00 min, with a final extension step of 60°C for 30 min. Genotyping was subsequently performed using an Applied Biosystems 3730 capillary analyser, and product lengths were determined relative to a GS500LIZ_3730 size standard. Fragment analyses were conducted using an ABI3730 XL DNA analyser. Microsatellite profiles were examined and scored manually and assessed for polymorphisms using GeneMapper version 4.0 (Applied Biosystems).

### 2.5 Population genetic analyses—remnants from a heavily fragmented landscape

Descriptive statistics were calculated for the microsatellite data using FSTAT ver. 2.9.3 (Goudet 1995) including (a) allelic richness per population averaged over loci; (b) Weir and Cockerham's inbreeding coefficient (*F*_IS_) and global population differentiation (*F*_ST_) with 95% confidence limits (Weir & Cockerham, 1984); (c) population pairwise measures of *F*_ST_ with significance determined using permutation (10,000); and (d) tests for linkage disequilibrium between loci using a log‐likelihood ratio test. The software Micro‐Checker ver. 2.2 (Van Oosterhout, Hutchinson, Wills, & Shipley, 2004) was used to assess microsatellite loci for null alleles and scoring errors using formula 1 outlined by Brookfield (1996), as evidence of null homozygotes was not apparent. We applied the FDR procedure when performing multiple simultaneous comparisons (Benjamini & Hochberg, 1995).

Estimates of observed (*H*_O_) and expected (*H*_E_) heterozygosity were determined using the Excel Microsatellite Toolkit (Park, 2001), and deviations from Hardy–Weinberg equilibrium (HWE) were determined using GENEPOP ver. 3.4 (Raymond & Rousset, 1995). Mean allelic richness and observed heterozygosity were compared among sample sites using a two‐sided permutation test (10,000 permutations), also implemented in FSTAT. An analysis of molecular variance (AMOVA) was performed in GenAlEx using pairwise *F*_ST_ as the distance measure, with 10,000 permutations and missing data for loci set at 10%. Identical multi‐locus genotypes were identified using the multi‐locus matching tool also implemented in GenAlEx.

Isolation by distance (IBD) analyses were performed to explore relationships between genetic differentiation and geographic distance between sites. Pairwise *F*_ST_ values were linearized (using Slatkin's linearized *F*_ST_ transformation (*F*_ST_/(1 − *F*_ST_)) and regressed to the natural log of geographic distance between sites (Rousset, 1997), with statistical significance evaluated by regression and Mantel testing using GenAlEx (Peakall & Smouse, 2006). Significance of Mantel tests was determined by permutation (10,000 randomizations).

A discriminant analysis of principal components (DAPC) was implemented in the *adegenet* package in R (Jombart, 2008; Jombart & Ahmed, 2011) to obtain a graphical depiction of patterns of genetic structure. The number of genetic clusters was then defined using k‐means, a clustering algorithm that looks for the value of K that maximizes the variation between groups. The Bayesian information criterion (BIC) was calculated for *K* = 1–22, and the *K* value with the lowest BIC was selected as the optimal number of clusters. A discriminant analysis was then performed using the function DAPC, implemented in R, to describe the genetic clusters.

The software package BOTTLENECK was used to test for evidence of recent reductions in the effective population size based on a comparison of allele numbers and observed heterozygosity at polymorphic loci (Piry, Luikart, & Cornuet, 1999). BOTTLENECK tests were performed on all remnants using the infinite allele model (IAM), stepwise mutation model (SMM) and the two‐phased model of mutation (TPM), with the intermediate TPM considered most suitable for microsatellite loci (Piry et al., 1999). The variance for TPM was set to 30% and the proportion of SMM in TPM set to 70%. Due to the relatively small number of loci, Wilcoxon's signed‐rank test was applied to determine significance based on 1,000 iterations. A qualitative descriptor of the allele frequency distribution (“mode‐shift” indicator), which discriminates bottlenecked populations from stable populations, was also calculated in BOTTLENECK. To account for multiple comparisons, we applied the FDR procedure (Benjamini & Hochberg, 1995).

Finally, relationships between individuals at each site were estimated with the program ML‐Relate (Kalinowski, Wagner, & Taper, 2006). ML‐Relate calculates coefficients of relatedness (*r*) and putative relationships among individuals (e.g. unrelated, siblings, parent/offspring) using a maximum‐likelihood approach.

### 2.6 Spatial autocorrelation analysis—remnants from less modified landscapes

Spatial autocorrelation analysis was performed in GenAlEx 6.501 to assess the spatial genetic structure of *B. marginata* at fine spatial scales among the Claude Austin Reserve, Victoria Valley and Durdidwarrah sampling replicates, providing a test of local genetic structuring and propagule movement and gene flow within unmodified landscapes. Distance classes for these analyses were based on the “equal sample size” option, with 9,999 permutations to test for levels of significance and using the “multi‐pop” test option. Spatial autocorrelation analysis was also replicated with SPAGEDI 1.2 (Hardy & Vekemans, 2002). We estimated the Queller and Goodnight (1989) relatedness coefficient among pairs of individuals belonging to the same a priori defined distance classes. For each class, random permutations in the spatial locations of individuals (10,000 permutations) were then used to assess deviations of the relatedness coefficient R from 0. Distance classes were chosen so that they contained more than 100 pairwise comparisons and had a participation index >50% and a coefficient of variation of participation of <1 (Hardy & Vekemans, 2002). Deviations from 0 mean that individuals within a given distance class are significantly more (positive values) or less (negative values) related than random. To assess the reliability of the results obtained with the Queller and Goodnight (1989) relatedness coefficient, we repeated the analyses using two other relatedness estimators, namely Lynch and Ritland’s (1999) relatedness coefficient (*r*) and the kinship coefficient of Loiselle, Sork, Nason, and Graham (1995).

### 2.7 Population simulations

Using microsatellite genotypic data (expected heterozygosity and allelic richness) generated for remnant *B. marginata* populations, we undertook simulations to predict the change in heterozygosity and average allele number over 200 generations based on effective population sizes (*N*_e_) of 20, 50, 100 and 500 (which are reflective of the distribution of current population size estimates for *B. marginata*). Simulations were undertaken in R Studio (version 1.2.1335) and loading libraries *psych* and *Rlab* (from www.r‐project.org). An R script was written to randomly sample two alleles with replacement at *x* loci for *N* number of individuals from a microsatellite data set (stored as a.csv file). The first step of the simulation created a panmictic population (*N* = 500, for *t* = 1) from a data set of observed genotypes from a population of *B. marginata*. We chose two populations to simulate as examples of a current population with high diversity (BBR, *H*_e_ *= *0.69*, A*_r_ *= *6.60) and a population with low diversity (BRS, *H*_e_ *= *0.42*, A*_r_ *= *3.10). A constant population size (e.g. *N* = 20, 50, 100 or 500) replicated 100 times was then created randomly (sampling with replacement) from the panmictic population of genotypes. These were then resampled independently for 200 nonoverlapping generations (*t*). At each generation, mean expected heterozygosity (*H*_e_) and allelic richness over loci (*A*_r_) were calculated as measures of genetic diversity.

We also undertook a simulation where we combined individuals from several populations to see the effect this had on genetic diversity through time for two constant population sizes (*N* = 250 and 400, reflective of a medium and relatively large population that would be the approximate target size for creating a new “mixed” population or, alternatively, connecting several remnant populations through ongoing assisted migration events). Genotypes from 20 individuals were randomly sampled from each of four populations (BRH, CRS, GLT and MOJ), all from a similar geographical area, creating a “mixed” population. Simulations were then run as above for *N* = 250 and 400, with *A*_r_ estimated at each generation. Confidence intervals (95%) for all simulations were calculated using the standard deviation from the 100 replicate populations at each *N* for each *t*.

### 2.8 Species distribution and climate niche modelling

The species distribution model for *B. marginata* was built in R (R Core Team, 2018) using boosted regression trees (Elith, Leathwick, & Hastie, 2008) using the gbm.step function, with dependencies from the *gbm* package (Ridgeway, 2017) and from the *dismo* package (Hijmans, Phillips, Leathwick, & Elith, 2017). A presence and pseudo‐absence data set (*n* = 4,052 comprising 2006 presences and 2046 absences) was developed using presence data from the Atlas of Living Australia (www.ala.org.au) and randomly generated pseudo‐absence point data. Presence data were filtered to choose only verified observations and datum with a spatial mapping accuracy of <500 m. This accuracy was chosen because we used climate data at 1‐km resolution. Presence and absence data points were only selected if they were >5 km apart to reduce spatial autocorrelation. The number of pseudo‐absence sites chosen was selected to ensure a balanced design of presence and pseudo‐absence points (Becker & Encarnacao, 2015). Species distribution models built with balanced presence and absence points are more accurate and less likely to contain biases that lead to model overfitting (Liu, Berry, Dawson, & Pearson, 2005; Mcpherson, Jetz, & Rogers, 2004). Climate data used in the analyses were from Worldclim 2 (Fick & Hijmans, 2017) with the exception of the aridity variable, annual heat moisture index, which was calculated from the mean annual temperature and precipitation bioclim variables (see Wang, Hamann, Yanchuk, O'Neill, & Aitken, 2006). Uncorrelated (*r* > .70) bioclim variables were selected (see Figure S3) for inclusion in the model, and the variables were as follows: mean annual temperature, maximum temperature of the warmest month, temperature seasonality, aridity, minimum temperature of the coldest month, mean temperature of the wettest quarter, precipitation seasonality and isothermality. Topography was considered using a topographic wetness index (Gallant & Austin, 2012) but was not included due to issues of scaling fine‐scale topographic processes at a ~30‐m resolution to the 1‐km resolution of the climate data. The BRT model was fit with a learning rate of 0.001, tree complexity of 5 and a bag fraction of 0.75 to ensure that the selected final model contained sufficient trees (>1,000) to yield a robust prediction. The data set was split into a calibration data set (75%) and a validation data set (25%). Model performance was assessed using the area under the receiver operating characteristic (AUC) (Fawcett, 2006), the Kappa statistic (Viera & Garrett, 2005) and deviance and cross‐validation deviance explained (Reside, VanDerWal, Moilanen, & Graham, 2017; Sutcliffe, Mellin, Pitcher, Possingham, & Caley, 2014). Climate change analyses used the ACCESS 1.0 model (Bi et al., 2013; Catto, Jakob, & Nicholls, 2013) and CMIP5 RCP 8.5 (Meinshausen et al., 2011) scenario projections for New South Wales, Victoria, South Australia and Tasmania provided by CSIRO and Bureau of Meteorology (2015). RCP 8.5 was chosen as it represents the worst‐case scenario for climate change in the region. We used the niche overlap analysis of Warren, Glor, and Turelli (2008) to calculate the spatial overlap of the distribution of the species climatic niche identified in the BRT modelling between current and future climates. The model probability that maximized model fit based on the Kappa statistic was selected as the threshold for determining the species climatic niche for this analysis.

## 3 RESULTS

### 3.1 Population genetic analyses—remnants from fragmented landscapes

A total of 497 individual *B. marginata* samples from 22 remnant populations were genotyped at 10 microsatellite loci (Tables 1 and S2). Marker independence was confirmed across all sample sites, with linkage disequilibrium analyses indicating no significant linkage between loci, while Micro‐Checker analyses found no evidence of scoring errors or null alleles at any locus. Identical multi‐locus genotypes were recorded among individuals from nine populations (BRH, BRS, CCR, DDK, FLB, HDB, KAL, MCL and SHF) with frequencies ranging from 1 to 13 identical genotypes (Table 2). As samples with identical genotypes are expected to represent root suckers, we included only one of the identical multi‐locus genotypes for analytical purposes. The inclusion of identical “root sucker” genotypes violates the expectation that estimates of population structure are derived from the comparison of allele frequencies from random samples of different outcrossing individuals at each sample location. The inclusion of identical “root sucker” genotypes is expected to skew the estimated allele frequencies within populations and potentially overstate the genetic uniqueness between populations. A total of 118 alleles were detected, with a mean of 4.65 alleles per locus across all sites (Table 2). Expected heterozygosity was moderate to high, ranging from 0.42 to 0.72 (mean *H*_E_ = 0.61).

**TABLE 2:** Statistics for 22 *Banksia marginata* collection sites screened with 10 microsatellite loci

Site	Site code	*n* clones	Relatedness	*A*	*r*	*H*_E_	*H*_O_	HWE	*F*_IS_
Bluebridge Road Elaine	BBR	0	87, 9, 2, 2 (13)	6.60	3.76	0.69	0.66	0.02	0.038
Mt Duneed	BGR	0	80, 16, 2, 2 (20)	5.70	3.70	0.70	0.68	0.38	0.028
Branxholme Railside	BRH	1	75, 10, 3, 12 (25)	5.20	3.11	0.63	0.72	0.67	−0.15
Ballan Railside	BRS	4	67, 6, 12, 15 (33)	3.10	2.11	0.42	0.50	0.38	−0.19
Cape Clear Rokewood Rd	CCR	2	78, 9, 4, 9 (22)	5.33	3.31	0.62	0.72	0.94	−0.16
Clarkefield railside.	CFR	0	75, 14, 4, 7 (25)	4.70	2.95	0.55	0.53	0.16	0.042
Caramut Roadside	CRS	0	79, 11, 4, 6 (21)	5.00	3.25	0.63	0.65	0.14	−0.029
Drik Drik	DDK	13	78, 13, 7, 2 (22)	4.11	3.22	0.70	0.73	0.09	−0.042
Durdidwarrah	DDW	0	81, 13, 2, 3 (18)	4.80	3.25	0.62	0.60	0.06	0.033
Dobie	DOB	0	76, 10, 7, 7 (24)	5.20	3.24	0.67	0.71	0.09	−0.053
Francis Lane	FLB	2	74, 10, 7, 9 (26)	3.30	2.54	0.49	0.57	0.71	−0.152
Glenthompson	GLT	0	77, 12, 3, 8 (23)	5.00	3.07	0.61	0.63	0.83	−0.024
Haddon	HDB	1	82, 11, 3, 4 (18)	4.60	3.00	0.61	0.63	**<0.001**	−0.031
Hawkesdale/Coltons Road	HWK	0	82, 10, 3, 5 (18)	4.30	2.85	0.56	0.60	0.82	−0.086
Kayleys Lane	KAL	6	87, 0, 13, 0 (13)	3.40	2.97	0.60	0.69	0.10	−0.168
Mount Clay	MCL	10	84, 4, 7, 5 (16)	4.30	2.94	0.64	0.65	0.08	−0.01
Moutajup	MOJ	0	80, 8, 3, 9 (20)	4.00	2.66	0.59	0.62	0.29	−0.069
Purdeet Siding	PDS	0	70, 10, 10, 10 (30)	2.70	2.29	0.48	0.56	0.99	−0.204
Pastoria East	PSE	0	67, 13, 7, 13 (33)	4.60	3.24	0.55	0.48	**<0.001**	**0.139**
St Helens Flora Reserve	SHF	1	91, 7, 0, 2 (9)	6.70	3.67	0.72	0.71	0.49	0.011
Skipton Rail Trail	SRT	0	83, 11, 2, 4 (17)	4.70	3.58	0.68	0.70	0.61	−0.043
Trawalla East	TRW	0	82, 10, 2, 6 (18)	5.10	3.58	0.69	0.63	0.72	0.139

Note

*n clones* = number of identical multi‐locus genotypes (potential clones/suckers) per site; *Relatedness* = values representing the percentage of unrelated, half‐sibling, full sibling and parent–offspring relationships (respectively) per site, with values in parentheses representing the total percentage of related individuals. Mean values averaged across loci for number of alleles (*a*), allelic richness (*r*), expected (*H*_E_) and observed (*H*_O_) heterozygosity, Hardy–Weinberg equilibrium *p*‐values (HWE) and inbreeding coefficients (*F*_IS_). Statistical significance (*α* = .05) after correction for multiple comparisons is indicated by bold text.

All sites were found to conform to HWE suggesting random mating (Table 2), except for sites HDB and PSE which showed significant deviations (*p* < .01; significant after corrections for multiple comparisons). For HDB, this estimate was influenced by a single locus only, while significant deviations were recorded at multiple loci for PSE. The estimate for PSE was supported by a significant inbreeding coefficient (*F*_IS_ = 0.139; *p* < .01), and a high estimate of relatedness among individuals. This site was unique in being dominated by many relatively small individuals among very few larger plants, and it is likely that many of the plants sampled are siblings from a small number of recruitment events.

Relatedness analyses revealed that a significant number of individuals at each collection site are direct relatives, with an average of 21% of samples being half‐ or full siblings. The lowest number of related individuals was recorded at SHF (9%) and the highest at BRS and PSE (33%) (Table 2).

A global estimate of *F*_ST_ across all loci was significantly different from zero (*F*_ST_ = 0.139; 95% confidence interval (CI) = 0.128–0.152) indicating genetic structure due to limited gene flow between sampling sites. Pairwise population comparisons of *F*_ST_ indicated significant genetic differentiation between all site pairs (231 pairwise comparisons), except for four comparisons which were not significant and were associated with sites in close proximity in the south‐west of the study area (Table S3). AMOVA analyses also indicated a high level of genetic variation between sites (14%, *p* < .01).

BOTTLENECK analyses found evidence of recent reductions in effective population size (bottleneck events) in 12 of 22 *B. marginata* populations (BBR, BGR, BRH, CRS, DDK, DDW, DOB, GLT, HDB, KAL, PSE and TRW). Wilcoxon's signed‐rank test for heterozygote excess was significant after correction for multiple comparisons under the IAM and TPM models for most remnants, while significant excess was observed under all three models for remnants KAL and TRW only. In most cases, these results were further supported by evidence of mode shifts, suggesting that many *B. marginata* remnants are not in mutation‐drift equilibrium and have undergone significant reductions in effective population size in the recent past.

The Mantel test indicates no relationship between genetic distance and geographic distance among sites across the VVP sample distribution. Similarly, there was no relationship detected with regression analyses (Figure S1). Based on an analysis of genetic variation across all sites, there was no significant association between genetic differentiation and geographic distance, with the Mantel test showing a nonsignificant relationship between Slatkin's linearized *F*_ST_ and the natural log of geographic distance (Mantel *r* = .09, *p* = .26). Regression also showed no significant relationship (*R* ^2^ = .01, *p* > .05).

Patterns of population genetic differentiation are graphically depicted by DAPC analyses that retained 200 principal components, and the first two discriminant functions, capturing 80% of the total variance within the SNP data set, and with k‐means identifying 14 population clusters (Figure S2). When plotted across the *x*‐ and *y*‐axes, individuals from the BRH and FLB sites cluster separately from the main cluster, including all remaining remnant populations (Figure S3A). These findings are consistent with pairwise *F*_ST_ estimates which suggest these populations are the most genetically divergent remnant populations. Further analyses were performed following the removal of individuals from these two sites, with the subsequent DAPC plot providing evidence of further genetic structuring with centroids of the SRT, BRS, KAL, CRS and CFR clusters appearing to cluster individually and away from the main cluster consisting of individuals from all remaining sites (Figure S3B).

### 3.2 Spatial autocorrelation analysis—remnants from less modified landscapes

**FIGURE 2:**
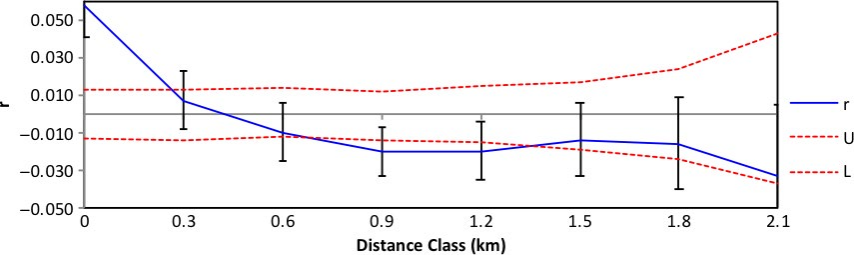
Spatial autocorrelation coefficient (*r*) for microsatellite data over a range of geographic distance classes for *Banksia marginata* sampled from Claude Austin Reserve, Victoria Valley and Durdidwarrah, with 95% confidence limits (U, L: upper and lower confidence limits, respectively)

A spatial autocorrelation analysis was performed using all unique multi‐locus genotypes (459 individuals) from *B. marginata* sampled at several sites separated by up to 2.2 km from the Claude Austin Reserve, Victoria Valley and Durdidwarrah. The relatedness coefficient (*r*) was calculated for all pairs of individuals, involving 6,260 pairwise comparisons across eight distance classes, ranging from 0 to 2,100 m. Significant and positive spatial autocorrelation was at 0 m (Figure 2), suggesting that individuals from the same collection sites are more genetically similar than would be expected at random. The autocorrelation signal becomes significantly negative at approximately 900 m, indicating that sites separated by these distances are more unrelated than expected if random mating was occurring throughout the sampling areas. This suggests a lack of gene flow among plants separated by >900 m.

### 3.3 Population simulations

**FIGURE 3:**
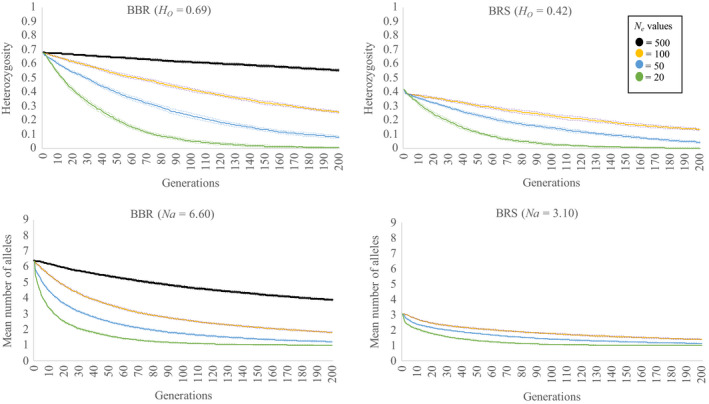
Simulations of *Banksia marginata* microsatellite data from the BBR (high genetic diversity) and BRS (low genetic diversity) remnants showing predicted change through time in expected heterozygosity (top), and mean number of alleles numbers (bottom) across ten microsatellite loci. The data are based on 1,000 simulations assuming random mating within the population and nonoverlapping generations. Dotted lines are 99% confidence intervals

The genetic simulations for the high and low diversity populations of *B. marginata* highlight how quickly genetic diversity is likely to be lost from populations if they are small (*N*_e_ ≤ 100) (Figure 3). At *N*_e_ ≤ 100, the high diversity population (BBR) loses half of its allelic diversity within 65 generations (for *N*_e_ = 20, only 12 generations) and half of its heterozygosity between 29 (*N*_e_ = 20) and 142 (*N*_e_ = 100) generations. Simulations with an *N*_e_ of ≥500 suggest the loss of genetic diversity over 200 generations is slowed. For the low diversity population BRS, simulations show that genetic diversity approaches fixation across loci within 200 generations when *N*_e_ = 20 or 50, and at *N*_e_ = 100, *H*_e_ = 0.14 and *A*_r_ = 1.41.

**FIGURE 4:**
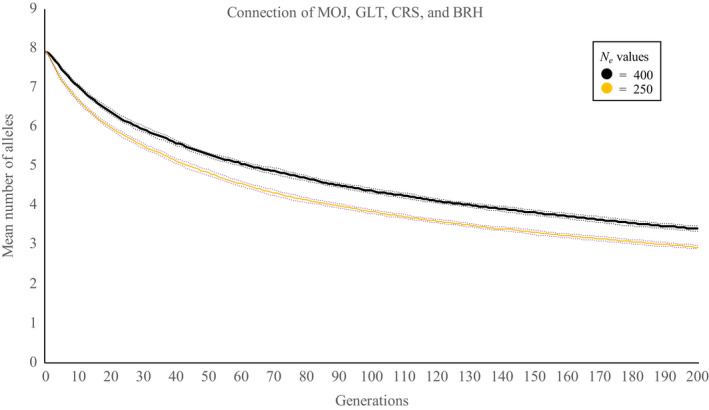
Simulations of *Banksia marginata* microsatellite data showing predicted change through time in mean number of alleles numbers (based on ten microsatellite loci) if populations MOJ, GLT, CRS and BRH are connected by assisted gene flow. The data are based on 1,000 simulations assuming random mating within the population and nonoverlapping generations. Dotted lines are 99% confidence intervals

We also simulated the consequences of mixing individuals from several different populations, a potential management action to stave off genetic decline. When individuals from several populations are mixed, genetic diversity can be increased substantially (Figure 4), and the loss of genetic diversity slowed if populations are able to be maintained at moderate, but practical *N*_e_ sizes (e.g. 240 or 400). We simulated combinations of individuals from four populations located in a similar region, and this resulted in a population with the highest allelic diversity (*A*_r_ = 7.9). However, over 200 generations, approximately half of the allelic diversity will still be lost at these effective population sizes (Figure 4).

### 3.4 Species distribution and climate niche modelling

**FIGURE 5:**
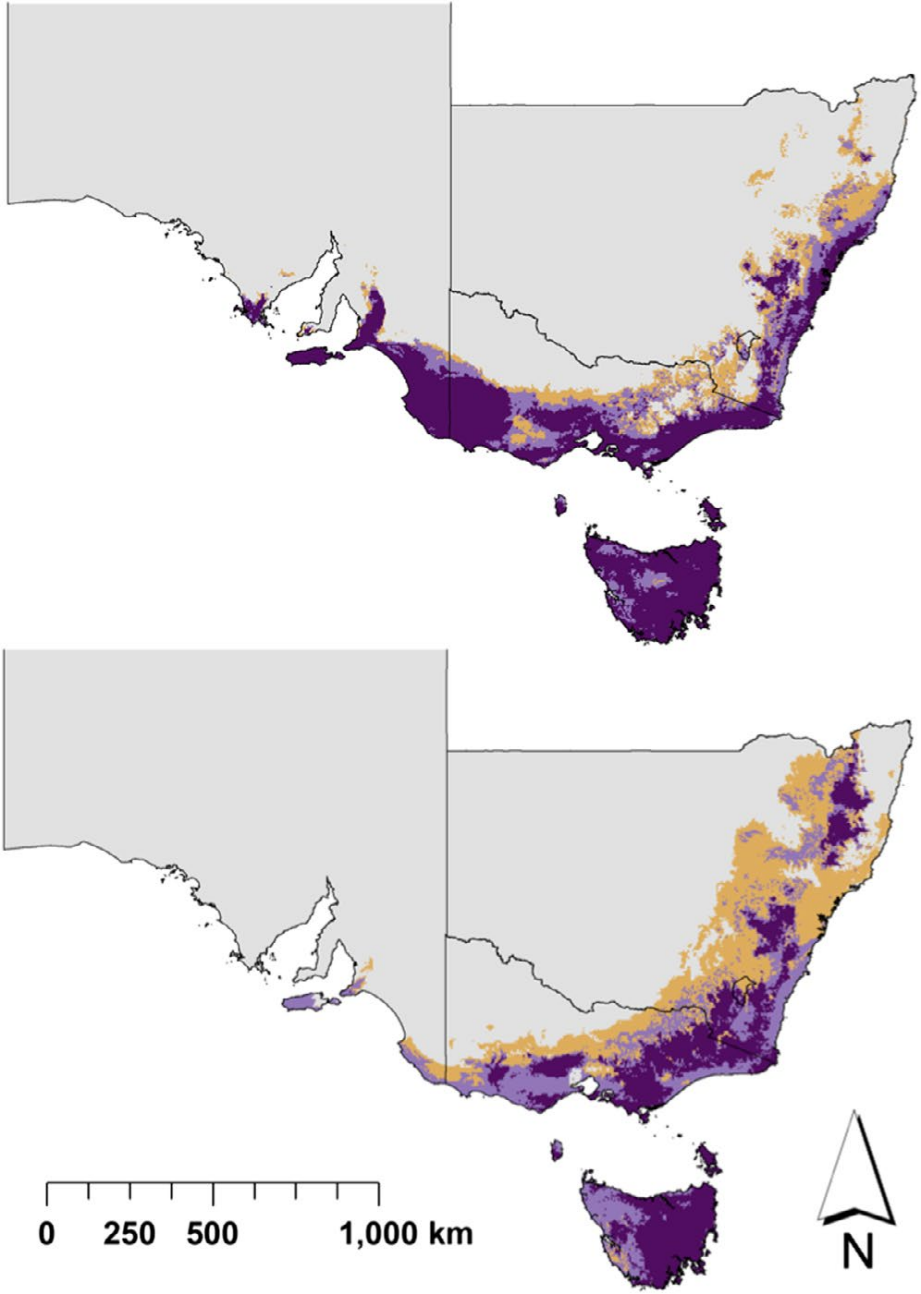
Predicted climatic niche for *Banksia marginata* in south‐eastern Australia under climate normal (top) and in the 2090s (bottom) based on RCP8.5 scenario and ACCESS 1.0 global circulation model. Grey areas are areas with high likelihood of absence, orange areas 20%–50% probability, light purple 50%–80% and dark purple >80% probability of occurrence, respectively

A robust model with excellent accuracy was developed (Table S4). Maximum temperature of the warmest month was the most influential variable for determining the climatic niche of *B. marginata* (Figure S4). Climate change was predicted to lead to a decline in suitability at 88% of the presence sites used to train the model, and a shift in the climatic niche to higher elevations with the current and future distribution of the species climate niche overlapping by 30%. Predicted distributions are illustrated in Figure 5. Climatic suitability, and change in suitability under climate change for all populations, is summarized in Table 3. Twenty of 25 populations were modelled to have climate less suitable for them by the 2080s. Declines in climatic suitability, based on the change in probability of occurrence from the BRT model, at these population sites, ranged from −2.1% to −87.5%. At the other five population sites, climate suitability increased from 0% to 20%.

**TABLE 3:** Modelled climate suitability index (CSI) of sites based on climate normal and in the 2090s under RCP 8.5 climate change scenario (CSI‐CC)

Site	Site code	CSI	CSI‐CC	Δ‐CSI (%)
Fragmented sites				
Bluebridge Road Elaine	BBR	0.94	0.91	−3.2
Mt Duneed	BGR	0.91	0.78	−14.3
Branxholme Railside	BRH	0.97	0.68	−29.9
Ballan Railside	BRS	0.95	0.92	−3.2
Cape Clear Rokewood Rd	CCR	0.60	0.72	20.0
Clarkefield Railside	CFR	0.87	0.81	−6.9
Caramut Roadside	CRS	0.81	0.81	0.0
Drik Drik	DDK	0.98	0.75	−23.5
Durdidwarrah	DDW	0.94	0.92	−2.1
Dobie	DOB	0.71	0.24	−66.2
Francis Lane	FLB	0.80	0.86	7.5
Glenthompson	GLT	0.89	0.90	1.1
Haddon	HDB	0.91	0.89	−2.2
Hawkesdale/Coltons Road	HWK	0.88	0.83	−5.7
Kayleys Lane	KAL	0.85	0.77	−9.4
Mount Clay	MCL	0.97	0.58	−40.2
Moutajup	MOJ	0.98	0.86	−12.2
Purdeet Siding	PDS	0.92	0.87	−5.4
Pastoria East	PSE	0.76	0.30	−60.5
St Helens Flora Reserve	SHF	0.82	0.63	−23.2
Skipton Rail Trail	SRT	0.81	0.84	3.7
Trawalla East	TRW	0.85	0.87	2.4
Unmodified sites				
Durdidwarrah		0.94	0.92	−2.1
Claude Austin Reserve		0.96	0.12	−87.5
Victoria Valley		0.97	0.82	−15.5

Note

Proportion of change in CSI is summarized as Δ‐CSI.

## 4 DISCUSSION

Species responses to climate change will depend on their ability to shift their distributions to accommodate climate changes, or rely on standing genetic variation to adapt in situ*.* Within this context, we investigated the likely evolutionary trajectory of a much depleted but once functionally important Proteaceae species from south‐eastern Australia, *Banksia marginata.* Population genetic analyses indicate most tree‐form remnant populations to be at risk of inbreeding and maladaptation. We argue that the long‐term viability of remnant populations will depend on assisted migration and seed mixing strategies aimed at maximizing population connectivity, genetic diversity and environmental resilience. Our simulations of population dynamics suggest that mixing individuals from different populations can mitigate genetic decline. Climate niche models predict widespread reductions in climate suitability across the species' contemporary distribution, highlighting the need to prioritize restoration investments that bolster existing and establishing new populations in areas expected to sustain *B. marginata* into the future. If the shifts in climate niche are as great as predicted, ecological replacement with an analogous species might need to be considered in areas where the species can no longer persist, in order to preserve local environmental values and ecosystem function.

### 4.1 Evidence of genetic structuring among and within remnant populations

Population genetic analyses revealed strong genetic structuring among *B. marginata* remnants across the Victorian Volcanic Plains, a pattern which has also been reported for other *Banksia* species (Carthew, 1993; Coates, McArthur, & Byrne, 2015; Evans, Ladiges, Newbigin, & Ades, 2001; Llorens, Byrne, Yates, Nistelberger, & Coates, 2012). This region is highly fragmented due to land clearing and agricultural intensification over the last century, leaving remnant populations isolated in the resulting landscape (Lunt & Spooner, 2005). Evidence of significant reductions in effective population size in recent generations for many remnants suggests that contemporary drift processes are likely to be contributing to the observed patterns of genetic differentiation and potentially a lack of isolation by distance. However, evidence of spatial correlation within largely intact remnants suggests that gene flow is likely to be inherently limited in this species.

Evidence of local genetic structuring in tree‐form *B. marginata* probably reflects limited seed movement, which is expected given the seeds are large and lack adaptations for long‐distance dispersal. Such patterns are also reported by Krauss et al. (2009) where seed dispersal in *B. hookeriana* averaged 5 m from the parent plant. Our results indicate that gene flow is unlikely to be exceeding 1 km in *B. marginata*. These findings are inconsistent with other studies on *Banksia* which have shown shallow levels of genetic differentiation and a capacity for long‐distance pollen dispersal due to mobile bird pollinators (Coates, Sampson, & Yates, 2007; Frick, Ritchie, & Krauss, 2014; Krauss et al., 2009; Ritchie & Krauss, 2012; Ritchie, Nevill, Sinclair, & Krauss, 2017). However, Llorens et al. (2012) demonstrated that the majority of matings determined by bird pollination within populations of *B. sphaerocarpa* var*. caesia* occurred between plants <60 m apart and, in most cases, <20 m apart, with only a small proportion of matings occurring at distances >400 m. Similarly, Ritchie, Dyer, Nevill, Sinclair, and Krauss (2019) demonstrated that while there appears to be a lack of genetic structure among *B. menziesii* populations separated by 10 s of kilometres, overall pollen dispersal appeared to be largely limited to distances <1.7 km. While further genetic surveys across additional large remnant habitats would help validate our findings, we suggest that gene flow may be inherently limited in *B. marginata*. A lack of gene flow raises questions about the potential factors influencing spatial patterns of pollination in *B. marginata*, highlighting the need for further studies into the synchrony of plant phenology among remnants, pollen viability, pollinator behaviour and landscape factors influencing pollinator movement.

### 4.2 Risks of inbreeding and the need for assisted migration

We found that many remnant tree‐form populations of *B. marginata* across the VVP are vulnerable to inbreeding due to their small and isolated nature. Our analyses indicate a high frequency of related individuals within isolated remnant stands, with an average of 21% of sampled individuals being direct relatives. It is uncertain whether local populations are already suffering from inbreeding effects or whether the species can tolerate some level of inbreeding, as this was not explicitly assessed in this study. However, evidence for recruitment bottlenecks in small remnant populations, with some populations failing to set seed (Heyes et al., 2019), suggests possible fitness reductions across at least part of the species distribution.

Population genetic simulations performed in this study indicate that most remnants across the VVP are likely to experience steep declines in genetic diversity as a result of inbreeding and random genetic drift. Our simulations demonstrate that the more genetically diverse remnants, with an *N*_e_ of 20, are likely to experience a 55% reduction in average allele numbers and 31% reduction in heterozygosity in the next 15 generations in the absence of intervention. This is a concern as the majority of *B. marginata* remnant populations spanning the VVP, and across much of its distribution, persist as small stands consisting of <20 individuals in total (Heyes et al. in review). In many cases, *N*_e_ is likely to be significantly lower than the census population size meaning that declines in genetic diversity will occur more rapidly (Frankham, Ballou, & Briscoe, 2010). While a number of factors can potentially skew the simulated rate of genetic erosion (i.e. overlapping generations, progeny backcrossing with parents, fitness reductions associated with inbreeding), our simulations indicate that declines in genetic variation across most remnant populations are imminent.

The simulations demonstrate the influence of *N*_e_ on risks of genetic erosion, with larger populations being less susceptible to drift processes (Figure 5). While our simulations indicate that an *N*_e_ of ≥500 is needed to ameliorate risks of genetic erosion, a value consistent with the literature (Frankham, Bradshaw, & Brook, 2014; Franklin, 1980; Jamieson & Allendorf, 2012), the potential to grow or maintain local populations to this size is often not possible given the constraints of existing agricultural land use. The simulations suggest that connecting remnant populations via assisted migration (i.e. translocation of seedlings or seed mixing) could be one way to achieve a larger effective population size and help overcome this problem (Figure 4).

Evidence suggests that minimal assisted migration (as little as one effective migrant contributing in successive generations) can provide adequate gene flow to connect populations, slow the loss of genetic diversity, decrease genetic load and allow differences in allele frequencies between populations to persist (Frankham et al., 2017; Weeks et al., 2011). The adoption of such approaches for conservation purposes has gained significant momentum over the last decade and provides a logical and logistically simple solution for connecting populations and overcoming risks of genetic erosion in highly fragmented landscapes. As genetic diversity is positively associated with population fitness in sexually reproducing species (Leimu, Mutikainen, Koricheva, & Fischer, 2006; Reed & Frankham, 2003), we argue that assisted migration will be an essential management tool for enhancing the long‐term viability and resilience of *B. marginata* remnants.

### 4.3 *Banksia* in a rapidly changing climate

Aside from risks associated with the genetic integrity of remnant tree‐form *B. marginata* populations, species distribution models suggest that the climatic niche for *B. marginata* will likely shift towards the eastern coast and higher elevations in Victoria and New South Wales and nearly disappear from South Australia. Of the 2006 presence observations selected, 88% of them were modelled to decline in climatic suitability, suggesting that populations may be under increased climatic stress in the future and at higher risk of population declines (Worrall et al., 2013). A key caveat to the modelled change in climatic suitability is that the model was population agnostic and, therefore, may not reflect the sensitivity or tolerance of different populations to climate change (i.e. potential influences of plastic or locally adapted traits). For example, Wang et al. (2006) found that populations of *P*. *contorta* had different temperature–productivity relationships that would result in different responses to climate change. Blake and Hill (1996) found that populations of *B. marginata* in Tasmania had significantly different frost and drought tolerances from mainland Australian populations which likely has facilitated the species' ability to occupy a wide climatic niche. For *B. marginata*, the response of individual populations to summer temperature—in terms of growth, reproduction and survival—is important consideration for making decisions about where to invest in *B. marginata* restoration, and for understanding spatial and temporal decline in areas that are likely to become climatically unsuitable in the coming decades.

Despite the potential limitations of SDMs (Nitschke & Innes, 2008; Sinclair, White, & Newell, 2010), these provide important tools for identifying areas within a species' range that may be vulnerable and areas outside of the current distribution that may be suitable for assisted migration or colonization (Ledig et al., 2012). Such interpretations should always be made with consideration of soils and terrain, which may further constrain suitable habitat. Based on SDMs produced by Rehfeldt and Jaquish (2010) for *Larix occidentalis*, British Columbia forest policy now permits the planting of this species in climatically suitable areas outside its current range (i.e. assisted range expansion) (Williams & Dumroese, 2013). SDMs can include population differences in fitness and productivity traits as predictors, or be developed for singular populations, to identify areas where provenances that are more climatically suitable to future conditions could be planted today, or in the near future, to facilitate species persistence under climate change (Rehfeldt & Jaquish, 2010). Williams and Dumroese (2013) termed this assisted population migration which is currently being practiced in some jurisdictions in North America in anticipation of future climate change.

The SDM has identified *B. marginata* populations that face significant changes in climatic suitability. Determining whether there is phenotypic and genotypic variation in tolerance to drought and heat stress in these populations is a critical next step for identifying the adaptive capacity within these populations and guiding future restoration efforts (Rice & Emery, 2003). Provenance trials are being established at various sites across the state of Victoria for *B. marginata* using seed from local and climate‐matched provenances (Figure S5), and glasshouse trials are underway, testing the relative performance of provenances sampled from across aridity gradients to drought and temperature stress. These trials will help to characterize patterns of local adaptation, and identify “climate‐ready” seed sources that can be used to enhance the resilience of populations at risk of maladaptation under climate change (Browne, Wright, Fitz‐Gibbon, Grugger, & Sork, 2019; Prober et al., 2015).

Areas of predicted future climate suitability from the SDM could be used to select sites for assisted range expansion. This might be considered if in situ conservation efforts to maintain *B. marginata* within its current distribution begin to fail and/or to proactively assist the species to adjust its distribution to keep track with the current velocity of climate change (Loarie et al., 2009). Given the predicted decline in climate suitability across much of the species range, ecological replacement of *B. marginata* using climatically suited plant species may be needed to maintain ecosystem processes where *B. marginata* cannot persist. From a functional perspective, the best option would be to replace *B. marginata* with another *Banksia* species that is adapted to drier and warmer environments. Lamont and Connell (1996) quantified the occurrence of 60 Banksia species across a climatic gradient in south‐west Australia. They identified a group of *Banksia* species that occur in areas with annual rainfall as low as 250 mm and mean maximum temperatures of the warmest month of 34.5°C. We found mean maximum temperature of the warmest month as the most influential variable for predicting *B. marginata's* distribution with an upper threshold of ~27°C (lower of ~22°C). The climate envelope of some *Banksia* species from south‐western Australia could therefore provide functional equivalence in areas of *B. marginata's* current range that become unsuitable in the future.

## 5 CONCLUSION

Taken together, our results suggest that securing the future of *B. marginata* will require significant interventions. This paper provides the data required to guide these interventions, highlighting the importance of enhancing the size, connectivity and genetic basis of remnant populations to help overcome risks of inbreeding and maladaptation. Populations must be maintained or created in areas with climates that remain suited to the species, and assisted migration to climatically suitable areas outside its current range may be needed in the future to help safeguard the species. As *B. marginata* is already showing signs of climate stress, seed mixing approaches that broaden the genetic basis of restoration plantings and include genotypes that will allow for adaptation to future climatic conditions will become increasingly important. Without such interventions, *B. marginata* is unlikely to persist in VVP region, and potentially other regions spanning its contemporary distribution, given the legacy of past demographic declines due to habitat fragmentation.

## CONFLICT OF INTEREST

None declared.

## AUTHOR CONTRIBUTIONS

This project was conceived by A.D.M, W.W, A.S and J.W.M, with all authors contributing to the study design. Field populations were identified and sampled by S.H, S.S. and W.W. A.D.M and O.H were responsible for genotyping, with genetic analyses led by A.D.M and A.R.W with contributions from L.B, S.E.H and C.D.H.S. Geospatial analyses were led by C.N with assistance from A.D.M, J.W.M and S.S. Writing of the manuscript was led by A.D.M with assistance from all authors.

## Supporting information

Supplementary Material

## Data Availability

Genetic and geospatial data are publicly available in DRYAD, https://doi.org/10.5061/dryad.31zcrjdh4.
