# Supplementary material for: Genetic data and climate niche suitability models highlight the vulnerability of a functionally important plant species from south‐eastern Australia
Source: Evol Appl. 2020 Apr 17;13(8):2014–29. doi: 10.1111/eva.12958 (PMC7463319; doi:10.1111/eva.12958)
Supplement: Supplementary file 1 — Supplementary Material [file EVA-13-2014-s001.pdf]

SUPPLEMENTARY INFORMATION

Table S1. Sample locations, sites from each location, and sample sizes included in analysis of genetic spatial autocorrelation in *B. marginata* analysis from large intact habitats

Sample location	Latitude	Longitude	Sample size
Durdidwarrah (Brisbane Ranges)			
Site 1	-37.805	144.205	10
Site 2	-37.804	144.203	10
Site 3	-37.804	144.200	10
Site 4	-37.800	144.199	10
Site 5	-37.797	144.201	10
Site 6	-37.789	144.198	10
Site 7	-37.795	144.190	10
Site 8	-37.799	144.191	10
Claude Austin Reserve (Grampians National Park)			
Site 1	-37.241	141.945	10
Site 2	-37.242	141.941	10
Site 3	-37.243	141.939	10
Site 4	-37.245	141.936	10
Site 5	-37.246	141.933	10
Site 6	-37.248	141.930	10
Site 7	-37.249	141.927	10
Site 8	-37.250	141.925	10
Site 9	-37.252	141.922	10
Site 10	-37.253	141.919	10
Victoria Valley (Grampians National Park)			
Site 1	-37.603	142.321	10
Site 2	-37.604	142.321	10
Site 3	-37.605	142.321	10
Site 4	-37.600	142.322	10
Site 5	-37.607	142.324	10
Site 6	-37.610	142.324	10
Site 7	-37.614	142.324	10
Site 8	-37.615	142.320	10
Site 9	-37.615	142.313	10
Site 10	-37.624	142.330	10

Table S2. List of microsatellite loci used in the current study

Locus	Allelic range	# of alleles	Reference
BM11	192 - 207	5	This study
BM12	238 - 252	8	"
BM22	227 - 254	11	"
BM27	125 - 156	14	"
BM3	273 - 310	15	"
BM31	204 - 234	20	"
BM37	260 - 289	11	"
BH-B8	241 - 294	21	He *et al.* 2008
Bint05VIC	231 - 268	8	Fatemi *et al.* 2013
Bint07NED	174 - 183	5	Fatemi *et al.* 2014

Table S3. Pairwise estimates of F_{ST} (lower diagonal) and D_{est} (upper diagonal) between 22 *Banksia marginata* collection sites based on 10 microsatellite loci. Values shown in bold are insignificant after corrections for multiple comparisons (P > 0.05).

	BBR	BGR	BRH	BRS	CCR	CFR	CRS	DDK	DDW	DOB	FLB	GLT	HDB	HWK	KAL	MCL	MOJ	PDS	PSE	SHF	SRT	TRW
BBR	*	0.09	0.26	0.29	0.14	0.14	0.16	0.12	0.16	0.18	0.25	0.17	0.15	0.24	0.27	0.18	0.20	0.29	0.25	0.09	0.15	0.11
BGR	0.04	*	0.34	0.29	0.21	0.16	0.21	0.14	0.18	0.18	0.32	0.26	0.29	0.30	0.23	0.20	0.24	0.35	0.33	0.16	0.23	0.13
BRH	0.12	0.12	*	0.28	0.33	0.42	0.29	0.17	0.26	0.29	0.38	0.17	0.28	0.32	0.23	0.27	0.21	0.44	0.39	0.21	0.28	0.21
BRS	0.16	0.16	0.22	*	0.36	0.35	0.28	0.22	0.39	0.28	0.34	0.36	0.33	0.41	0.30	0.21	0.28	0.33	0.44	0.22	0.33	0.27
CCR	0.08	0.11	0.21	0.28	*	0.15	0.12	0.20	0.13	0.24	0.21	0.26	0.18	0.30	0.27	0.24	0.15	0.23	0.35	0.15	0.21	0.20
CFR	0.07	0.09	0.20	0.21	0.17	*	0.16	0.28	0.23	0.19	0.30	0.31	0.19	0.34	0.33	0.29	0.20	0.34	0.36	0.16	0.30	0.21
CRS	0.06	0.08	0.15	0.19	0.12	0.10	*	0.09	0.21	0.14	0.17	0.20	0.25	0.20	0.26	0.15	0.09	0.12	0.34	0.09	0.18	0.23
DDK	0.05	0.04	0.09	0.16	0.12	0.15	0.04	*	0.15	0.12	0.22	0.07	0.26	0.16	0.19	**0.06**	0.10	0.16	0.27	0.06	0.17	0.12
DDW	0.07	0.08	0.14	0.22	0.13	0.13	0.12	0.07	*	0.23	0.30	0.15	0.27	0.31	0.24	0.21	0.17	0.32	0.34	0.13	0.24	0.17
DOB	0.06	0.05	0.15	0.18	0.16	0.11	0.09	0.05	0.12	*	0.30	0.20	0.24	0.19	0.13	0.15	0.14	0.25	0.27	0.09	0.20	0.11
FLB	0.19	0.19	0.25	0.31	0.21	0.23	0.18	0.19	0.23	0.21	*	0.32	0.31	0.25	0.38	0.30	0.22	0.24	0.35	0.24	0.16	0.34
GLT	0.08	0.11	0.10	0.22	0.19	0.15	0.11	0.04	0.07	0.10	0.23	*	0.25	0.16	0.21	0.16	0.16	0.28	0.34	0.11	0.21	0.14
HDB	0.07	0.09	0.18	0.23	0.16	0.08	0.12	0.13	0.12	0.11	0.25	0.13	*	0.36	0.29	0.32	0.27	0.38	0.31	0.17	0.20	0.18
HWK	0.13	0.17	0.22	0.34	0.18	0.24	0.13	0.11	0.20	0.12	0.29	0.18	0.25	*	0.25	0.18	0.23	0.27	0.33	0.18	0.20	0.27
KAL	0.11	0.10	0.15	0.24	0.18	0.21	0.15	0.10	0.13	0.08	0.32	0.14	0.17	0.18	*	0.20	0.22	0.33	0.37	0.20	0.22	0.16
MCL	0.10	0.10	0.15	0.19	0.15	0.20	0.09	**0.03**	0.14	0.08	0.27	0.12	0.16	0.14	0.11	*	0.14	0.21	0.37	**0.04**	0.27	0.20
MOJ	0.09	0.09	0.10	0.19	0.15	0.13	0.07	0.05	0.11	0.11	0.18	0.08	0.15	0.16	0.17	0.11	*	0.18	0.38	0.09	0.24	0.24
PDS	0.18	0.18	0.27	0.34	0.18	0.27	0.13	0.12	0.23	0.18	0.23	0.20	0.26	0.26	0.26	0.16	0.18	*	0.50	0.20	0.29	0.38
PSE	0.12	0.14	0.23	0.30	0.24	0.20	0.18	0.15	0.16	0.15	0.29	0.18	0.17	0.23	0.20	0.20	0.24	0.33	*	0.32	0.27	0.25
SHF	0.03	0.05	0.10	0.13	0.10	0.09	0.06	**0.01**	0.07	0.04	0.17	0.04	0.09	0.13	0.10	**0.03**	0.05	0.12	0.16	*	0.17	0.13
SRT	0.06	0.08	0.16	0.20	0.13	0.13	0.08	0.08	0.09	0.08	0.15	0.11	0.11	0.16	0.12	0.13	0.12	0.19	0.15	0.06	*	0.18
TRW	0.05	0.04	0.12	0.18	0.12	0.11	0.12	0.05	0.08	0.05	0.22	0.07	0.10	0.20	0.08	0.09	0.11	0.19	0.14	0.04	0.10	*

Table S4. Goodness of Fit statistics for *B. marginata* species distribution model. AUC and KAPPA values indicate model has excellent accuracy (AUC) and agreement (KAPPA) with observed data respectively. Classification accuracy indicates the proportion of present and absent sites correctly identified.

Total Deviance: Calibration (%)	Deviance: Cross Validation (%)	Total Deviance: Validation (%)	AUC	KAPPA	Classification Accuracy
81	71	85	0.97	0.85	0.92

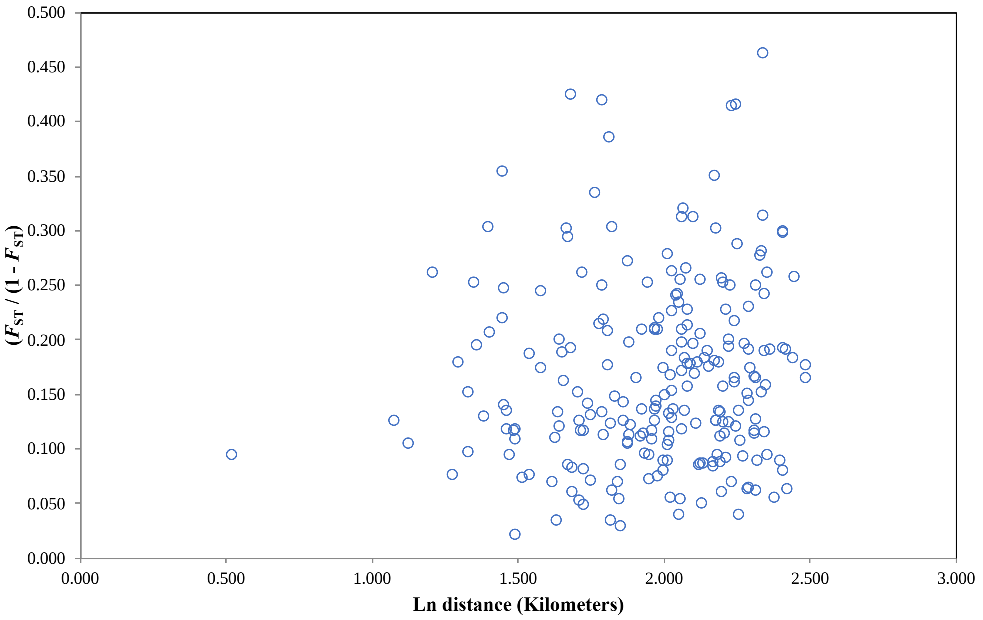

Figure S1. Regression analysis for the *B. marginata* microsatellite dataset, linearized F_{ST} against the natural log of the pairwise distance (in km), indicating no significant association between genetic differentiation and geographic distance (r = 0.09, R^2 = 0.008, P > 0.05).

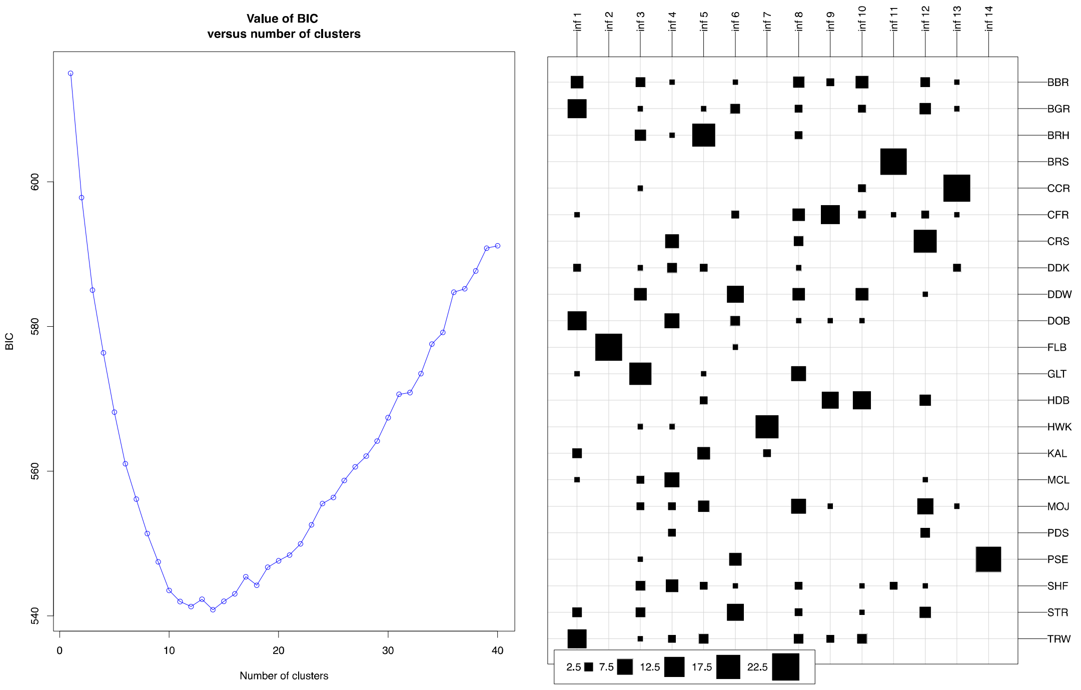

Figure S2. Discriminant analysis of principal components. Left panel; Bayesian Information Criteria (BIC) identifying 14 clusters. Right panel; population assignment to inferred population clusters (inf 1-14) indicated by black shaded boxes, where the boxes reflect relative proportions of membership.

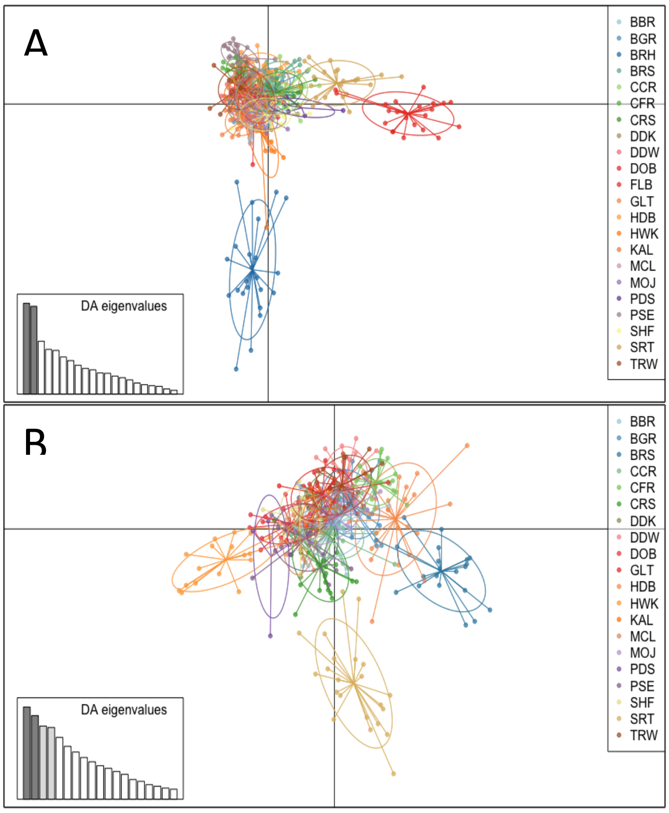

Figure S3. Discriminant Analysis of Principle Components; (A) including individuals from all 22 *B. marginata* remnants, (B) individuals from all remnants, excluding BRH and FLB.

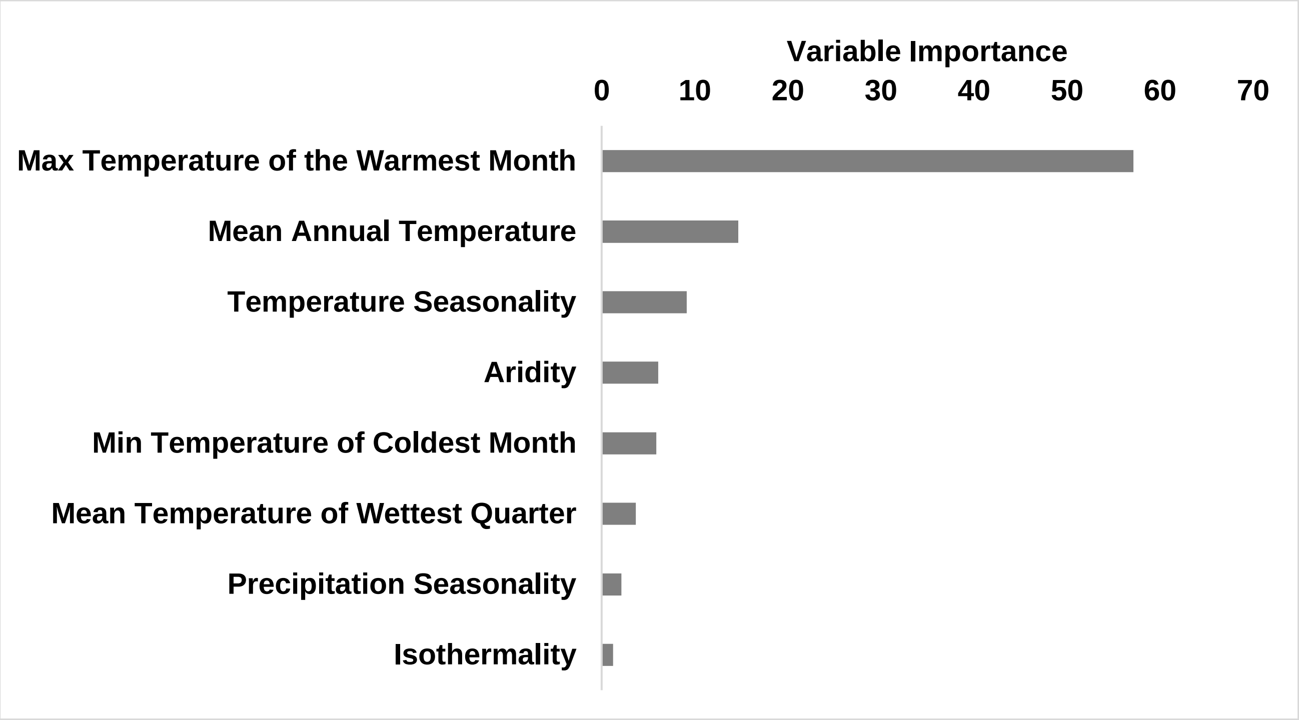

Figure S4. Relative influence of independent variables from boosted regression modelling for predicting climatic niche of *Banksia marginata*.

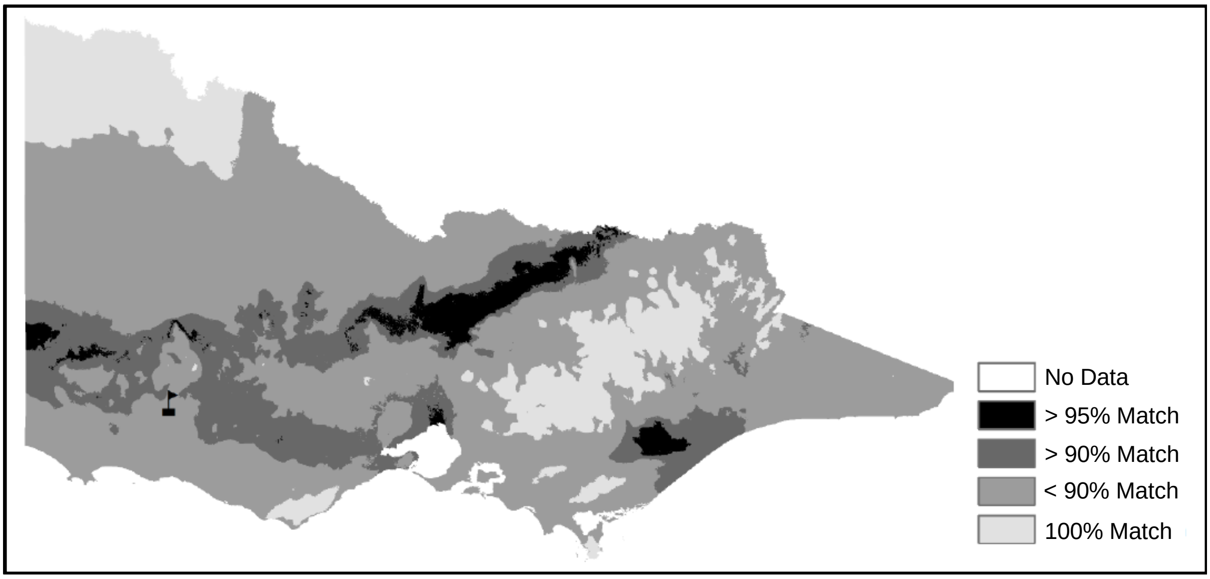

Figure S5. Climate matching for a provenance trial site at Dunkeld in southwestern Victoria (site indicated by flagged symbol). Climate matching based on 2080 RCP8.5 scenario and ACCESS 1.0 global circulation model, performed using annual climate data for Dunkeld. Areas with >95% climate match are regarded as having a current climate that matches the target site (suitable areas for climate matched seed sources).
